# Long-term combination chemotherapy using eribulin and trastuzumab for three patients with human epidermal growth factor receptor 2-positive metastatic breast cancer

**DOI:** 10.1007/s13691-016-0253-y

**Published:** 2016-06-21

**Authors:** Toshiaki Saeki, Kazuhiro Araki, Ken Shimada, Takashi Shigekawa, Hirofumi Nakayama, Yoshihiko Segawa, Hirofumi Mukai

**Affiliations:** 1 Department of Breast Oncology, International Medical Center, Saitama Medical University, 1397-1, Yamane, Hidaka, Saitama, 350-1298 Japan; 2 Breast Medical Oncology, Breast Oncology Center, The Cancer Institute Hospital of JFCR, 3-8-31, Ariake, Koto-ku, Tokyo, 135-0141 Japan; 3 Division of Medical Oncology, Department of Medicine, Showa University Koto Toyosu Hospital, 5-1-38, Toyosu, Koto-Ku, Tokyo, 135-8577 Japan; 4 Division of Medical Oncology, Department of Medicine, Showa University Fujigaoka Hospital, 1-30, Fujigaoka, Aoba-ku, Yokohama, Kanagawa 227-8501 Japan; 5 Department of Medical Oncology, International Medical Center, Saitama Medical University, 1397-1, Yamane, Hidaka, Saitama, 350-1298 Japan; 6 Department of Breast and Medical Oncology, National Cancer Center Hospital East, 6-5-1, Kashiwanoha, Kashiwa, Chiba 277-8577 Japan

**Keywords:** Breast cancer, Eribulin, Trastuzumab, Anticancer drug, Combination chemotherapy

## Abstract

The combination chemotherapy regimen of eribulin (ERI) and trastuzumab (TRA)—the ERI-TRA regimen—has been shown to be highly tolerable for patients with recurrent or metastatic human epidermal growth factor receptor 2 (HER2)-positive breast cancer. However, no sufficient clinical evidence is available for the long-term safety profile of the regimen. We report on three patients in the Phase I combination study of the regimen, for whom the regimen could be conducted over the long term. Patient #1 was a 68-year-old woman and underwent the regimen until cycle 23. Patient #2 was a 61-year-old woman and underwent the regimen until cycle 27. Patient #3 was a 59-year-old woman and underwent the regimen until cycle 22. All these patients had undergone TRA-based combination therapy before the onset of the regimen. Any new categories of adverse events did not occur in association with the long-term combination chemotherapy. Neutropenia experienced by these patients was reversible and easily manageable by dose adjustment (interruption/delay and reduction). Neither increase in the risk of cardiomyopathy nor the worsening of peripheral neuropathy greater than grade 1 was found. The present regimen was suggested to be a novel chemotherapeutic option for patients with HER2-positive recurrent or metastatic breast cancer. The fact that the long-term ERI-TRA regimen was successfully conducted for these patients can be supplementary clinical information that is beneficial for clinical oncologists.

## Introduction

We conducted a Phase I combination study of eribulin (ERI) mesylate and trastuzumab (TRA) in 12 Japanese female patients with HER2-positive metastatic breast cancer [1]. The design of the study consisted of Part 1 (weekly administration of TRA) and Part 2 (triweekly administration of TRA). In both Part 1 and 2, ERI mesylate 1.4 mg/m^2^ [equivalent to ERI 1.23 mg/m^2^ (expressed as free base)] was administered intravenously for 2–5 min on days 1 and 8 of each cycle. In Part 1, TRA 4 mg/kg was given by ≥90-minute infusion as the loading dose, followed by ≥30-minute infusion at 2 mg/kg on days 1, 8, and 15 of the 21-day cycle. In Part 2, TRA 8 mg/kg was administered as the loading dose, followed by infusion at 6 mg/kg on day 1 of the second and subsequent 21-day cycles [1]. Consequently, the combination chemotherapy regimen of ERI mesylate 1.4 mg/m^2^ on days 1 and 8 of the 21-day cycle with weekly or triweekly TRA was shown to be highly tolerable. Results from the Phase I combination study [1] and the Phase II study [2] suggest the reasonableness of further examining the long-term safety profile of the regimen.

We hereby report on three Japanese patients with HER2-positive metastatic breast cancer for whom this regiment was continued after the cutoff date (August 2, 2013) in the Phase I combination study and in whom subsequent 9-month follow-up revealed that the total number of the chemotherapy cycles had exceeded 20.

## Case reports

Patient #1: A 68-year-old woman in Part 1. The patient had undergone mastectomy for right breast cancer at our hospital and was enrolled in this study because of progressive disease (PD). Immunohistochemistry (IHC) staining revealed HER2 positivity (3+), as well as estrogen receptor (ER) and progesterone receptor (PgR) negativity. The patient had been treated with a combination of tegafur and tamoxifen as anticancer drugs for adjuvant chemotherapy, followed by a combination of TRA and paclitaxel (PTX) for the treatment of breast cancer that recurred. At baseline, the patient had grade 1 peripheral neuropathy but did not present cardiac dysfunction. After enrollment, dose-limiting toxicity (DLT) did not develop. The unequivocal progression of nontarget lesions in the right femur led to treatment discontinuation in cycle 23. Grade 2 or greater adverse events (AEs) in this study included grade 4 neutropenia, grade 3 leukopenia, grade 2 mucositis, grade 2 phlebitis, grade 2 anorexia, grade 2 dysgeusia, and grade 2 alopecia. Reduced left ventricular ejection fraction (LVEF) was not found.

Patient #2: A 61-year-old woman in Part 1. The patient had undergone conservation surgery of the left breast at our hospital and was enrolled in this study because of PD. IHC staining revealed ER and PgR negativity, as well as HER2 positivity (2+) that was subsequently verified by gene amplification by fluorescence in situ hybridization (FISH). The patient had been treated by combination chemotherapy with doxorubicin (DXR) and cyclophosphamide, followed by PTX alone, radiotherapy, and TRA alone. Furthermore, the patient underwent combination chemotherapy with TRA and docetaxel (DTX) for the treatment of breast cancer that recurred. At baseline, the patient had grade 1 peripheral neuropathy but did not present cardiac dysfunction. After enrollment, DLT did not develop. The unequivocal progression of nontarget lesions in the right kidney led to treatment discontinuation in cycle 27. Grade 2 or greater AEs in this study included grade 4 neutropenia, grade 2 leukopenia, grade 2 extravascular leakage at injection site, grade 2 LVEF, grade 2 alopecia, and grade 2 hyperlipidemia. Grade 2 LVEF developed in cycle 19.

Patient #3: A 59-year-old woman in Part 2. The patient could not be treated surgically because of stage IIIc breast cancer and was referred to our hospital for enrollment in this study. IHC staining revealed ER and PgR positivity, as well as HER2 positivity (3+) that was subsequently verified by FISH. The patient had been treated by combination chemotherapy with TRA and PTX, followed by TRA alone. At baseline, the patient had grade 1 peripheral neuropathy but did not present cardiac dysfunction. After enrollment, DLT did not develop. Grade 2 or greater AEs in this study included grade 4 neutropenia, grade 3 febrile neutropenia, grade 3 hypertriglyceridemia, grade 2 leukopenia, grade 2 lymphopenia, grade 2 anemia, grade 2 pyrexia, grade 2 spondylolisthesis, grade 2 stomatitis, and grade 2 urticaria. Treatment was discontinued due to interstitial pneumonia in cycle 22. Reduced LVEF was not found. Patient characteristics at baseline are shown in Table 1.

**Table 1:** Patient characteristics at baseline

Variables	Patients		
	*N* = 3*	*N* = 9^†^	Total = 12
ER and/or PgR plus disease, *n* (%)	1 (33.3)	4 (44.4)	5 (41.7)
ECOG performance status, *n* (%)			
0	0	5 (41.7)	5 (41.7)
1	3 (100)	4 (44.4)	7 (58.3)
Number of prior chemotherapy regimens, *n* (%)^‡^			
1	1 (33.3)	0	1 (8.3)
2	0	1 (11.1)	1 (8.3)
3	2 (66.7)	0	2 (16.7)
4	0	2 (22.2)	2 (16.7)
≥5	0	6 (66.7)	6 (50.0)
Median (range)	3.0 (1–3)	5.0 (2–14)	4.5 (1–14)
Number of prior trastuzumab treatment, *n* (%)^‡^			
1	2 (66.7)	1 (11.1)	3 (25.0)
2	1 (33.3)	3 (33.3)	4 (33.3)
≥3	0	5 (55.6)	5 (41.7)
Median (range)	1.0 (1–2)	3.0 (1–5)	2.0 (1–5)
Prior taxane treatment, *n* (%)^‡^			
1	2 (66.7)	5 (55.6)	7 (58.3)
2	1 (33.3)	3 (33.3)	4 (33.3)
3	0	1 (11.1)	1 (8.3)
Prior anthracycline treatment, *n* (%)	1 (33.3)	5 (55.6)	6 (50.0)

*ECOG* Eastern Cooperative Oncology Group, *ER* estrogen receptor, *PgR* progesterone receptor

* Patients who received more than 20 cycles of the treatment

^†^Patients who received less than 20 cycles of the treatment

^‡^Including the neoadjuvant, adjuvant, and therapeutic regimens

The Phase I combination study described two patients with grade 2 reduced LVEF and indicated no clearly increased risk of severe cardiac dysfunction resulting from the present combination chemotherapy regimen because they recovered within 1 week without requiring treatment. The follow-up subsequent to the cutoff date for the Phase I combination study did not disclose any new case of reduced LVEF (Table 2).

**Table 2:** Adverse events (all grades in ≥10 % of patients and grade 3/4 in total)

Preferred terms in CTCAE, *n* (%)	Patients					
	*N* = 3*		*N* = 9^†^		Total	
	All grades	Grade 3/4	All grades	Grade 3/4	All grades	Grade 3/4
Leukopenia	3 (100.0)	2 (66.7)	9 (100.0)	8 (88.9)	12 (100.0)	10 (83.3)
Neutropenia	3 (100.0)	3 (100.0)	9 (100.0)	9 (100.0)	12 (100.0)	12 (100.0)
Anemia	2 (66.7)	0	6 (66.7)	0	8 (66.7)	0
Lymphopenia	1 (33.3)	1 (33.3)	2 (22.2)	2 (22.2)	3 (25.0)	3 (25.0)
Febrile neutropenia	1 (33.3)	1 (33.3)	1 (11.1)	1 (11.1)	2 (16.7)	2 (16.7)
Palpitations	1 (33.3)	0	1 (11.1)	0	2 (16.7)	0
Nausea	0	0	3 (33.3)	0	3 (25.0)	0
Vomiting	1 (33.3)	0	2 (22.2)	0	3 (25.0)	0
Constipation	1 (33.3)	0	1 (11.1)	0	2 (16.7)	0
Stomatitis	1 (33.3)	0	1 (11.1)	0	2 (16.7)	0
Pyrexia	2 (66.7)	0	3 (33.3)	0	5 (41.7)	0
Malaise	2 (66.7)	0	1 (11.1)	0	3 (25.0)	0
Chest discomfort	1 (33.3)	0	1 (11.1)	0	2 (16.7)	0
Injection site reaction	1 (33.3)	0	1 (11.1)	0	2 (16.7)	0
Lung infection	0	0	2 (22.2)	0	2 (16.7)	0
Tonsillitis	2 (66.7)	0	0	0	2 (16.7)	0
ALAT increased	1 (33.3)	0	2 (22.2)	0	3 (25.0)	0
ASAT increased	1 (33.3)	0	2 (22.2)	0	3 (25.0)	0
Blood CPK increased	1 (33.3)	0	2 (22.2)	0	3 (25.0)	0
Ejection fraction decreased^§^	1 (33.3)	0	1 (11.1)	0	2 (16.7)	0
Decreased appetite	3 (100.0)	0	3 (33.3)	0	6 (50.0)	0
Hypertriglyceridemia	1 (33.3)	1 (33.3)	2 (22.2)	2 (22.2)	3 (25.0)	3 (25.0)
Hypophosphatemia	0	0	1 (11.1)	1 (11.1)	1 (8.3)	1 (8.3)
Myalgia	2 (66.7)	0	2 (22.2)	0	4 (33.3)	0
Muscle spasms	1 (33.3)	0	1 (11.1)	0	2 (16.7)	0
Dysgeusia	2 (66.7)	0	2 (22.2)	0	4 (33.3)	0
Peripheral neuropathy^‡^	2 (66.7)	0	3 (33.3)	1 (11.1)	5 (41.7)	1 (8.3)
Headache	1 (33.3)	0	1 (11.1)	0	2 (16.7)	0
Alopecia	3 (100.0)	NA	5 (55.6)	NA	8 (66.7)	NA
Rash	2 (66.7)	0	3 (33.3)	0	5 (41.7)	0

*ALAT* alanine aminotransferase, *ASAT* aspartate aminotransferase, *CPK* creatinine phosphokinase, *NA* not applicable, *CTCAE* Common terminology criteria for adverse events, Japanese version 4.0

* Patients who received more than 20 cycles of the treatment

^†^Patients who received 20 or less cycles of the treatment

^‡^Including neuropathy peripheral and peripheral sensory neuropathy in preferred terms

^§^Patient #2 developed this change in cycle 19

## Discussion

At the onset of the Phase I combination study in 2011, the preferred first-line chemotherapy regimens recommended by the National Comprehensive Cancer Network (NCCN) [3]—which combined TRA with one and/or two anticancer drugs, e.g., PTX [4, 5], DTX [6–10], capecitabine [CAPE] [7, 11, 12], and vinorelbine [VNR] [10, 13, 14]—were conducted for patients with HER2-positive recurrent or metastatic breast cancer. The package inserts of these anticancer drugs describe the following as major AEs: (1) TRA: cardiomyopathy and anaphylactoid symptoms; (2) PTX: myelosuppression, hypersensitive reaction, and peripheral neuropathy; (3) DTX: myelosuppression, anaphylaxis, and nail disorders; (4) CAPE: dehydration, hand-foot syndrome, and hepatopathy; and (5) VNR: myelosuppression, interstitial pneumonia, pulmonary congestion, and phlebitis. Furthermore, myelosuppression, infections, peripheral neuropathy, and hepatic dysfunction have been reported regarding ERI.

Neutropenia experienced by all 12 patients in the Phase I combination study was reversible and easily manageable by dose adjustment (interruption/delay and reduction). Three of 12 patients, in whom the long-term ERI-TRA regimen could be conducted, showed no increased risk of cardiomyopathy in association with the present regimen; nevertheless, reduced LVEF was observed with two patients prior to the cutoff date. Therefore, cardiac function should be routinely assessed in patients undergoing the ERI-TRA regimen, which is consistent with the recommendation for patients receiving trastuzumab in other combination therapies. Nonhematologic AEs, e.g., nausea, vomiting, pyrexia, and rash, were mild in intensity. Especially, it is of note that this regimen provoked little AEs that considerably affect the quality of life of patients during the chemotherapy cycles, e.g., hypersensitive reaction, nail disorders, phlebitis, as well as hand-foot syndrome observed with PTX, DTX, VNR, and CAPE, respectively. Prior studies of PTX and DTX have reported the incidences of grade 3/4 peripheral neuropathy ranging between 5–35 % [15–17] and 3–14 % [18, 19]. Peripheral neuropathy was grade 1/2 in all 12 patients, except one who had grade 3 peripheral neuropathy. Furthermore, any peripheral neuropathy that worsened to greater than grade 1 was not found in these three patients. At baseline, on the other hand, the patients presented the less number of prior chemotherapy regimens and less exposure to TRA as compared with the remaining nine patients; these facts probably favored the long-term management of the pathology. Examination of the safety profile revealed no emergence of any new categories of AEs associated with long-term combination chemotherapy subsequent to the cutoff date of Phase I combination study, which leads us to consider that the present regimen is safe for a long term. Taken together, we believe that information on the long-term safety of eribulin described above is of clinical relevance for clinical oncologists.

The objectives of treatment for patients with recurrent or metastatic breast cancer, about whom cure is hopeless, are the extension of their survival and the sustainment of or improvement in their quality of life. Therefore, it is essential to conduct anticancer combination chemotherapy for a longer period of time to fulfill these objectives. In many clinical settings, however, we frequently find treatment difficult to continue because of disease progression, sorts of AEs, loss of efficacy, and others. In fact, the ERI-TRA regimen was discontinued not later than cycle 12 in nine of our 12 patients [1]. The 2015 NCCN Clinical Practice Guidelines in Oncology: Breast Cancer mention two preferred first-line regimens for chemotherapy combinations: pertuzumab (PER), TRA, and DTX; and PER, TRA, and PTX, in addition to other first-line regimens combining TRA with PTX ± carboplatin, DTX, CAPE, or VNR [20]. The results from our analysis indicated no deterioration of hematologic or nonhematologic AEs in three patients of interest, suggesting the potential of the present regimen as a novel chemotherapeutic option for patients with HER2-positive recurrent or metastatic breast cancer.

The fact that long-term chemotherapy using ERI mesylate in combination with TRA was successfully conducted for patients with recurrent or metastatic HER2-positive breast cancer can be supplementary clinical information that is beneficial for clinical oncologists.
